# How Patients Dispose of Unused Prescription Opioids: A Survey of over 300 Postoperative Patients

**DOI:** 10.7759/cureus.28111

**Published:** 2022-08-17

**Authors:** Ramin Talebi, Chaim Miller, Jack Abboudi, Shyam Brahmabhatt, William Emper, Jess Lonner, Justin Kistler, Donald Mazur, David Pedowitz, Asif M Ilyas

**Affiliations:** 1 Orthopaedics, Sidney Kimmel Medical College at Thomas Jefferson University, Philadelphia, USA; 2 Division of Hand Surgery, Rothman Orthopaedic Institute, Philadelphia, USA; 3 Division of Sports Medicine, Rothman Orthopaedic Institute, Philadelphia, USA; 4 Division of Knee Surgery, Rothman Orthopaedic Institute, Philadelphia, USA; 5 Division of Foot & Ankle Surgery, Rothman Orthopaedic Institute, Philadelphia, USA; 6 Foundation for Opioid Research & Education, Rothman Orthopaedic Institute, Philadelphia, USA

**Keywords:** prescription opioids, opioid use, orthopedic pain management, orthopedic surgery, opioid disposal

## Abstract

Introduction

Diversion of unused prescription opioids is a common source of opioid sensitization in the community. Educating patients about safe opioid use has been shown to be effective in decreasing opioid use. However, decreasing diversion will also require educating patients on proper opioid disposal. A survey was administered to better understand patients’ habits with opioid disposal for opioids prescribed after orthopedic surgery.

Methods

A cross-sectional survey study of 469 patients who had undergone orthopedic surgery was conducted to learn their preferences and habits regarding the disposal of unused prescription opioids received after orthopedic surgery.

Results

The survey respondents consisted of 48.8% female and 51.2% male patients. Ninety-four point two percent (94.2%) of those receiving opioid prescriptions reported having leftover unused opioids. In terms of voluntary disposal, 68.8% claimed to dispose of their prescription opioids while 31.2% did not. Gender, but not age, had a significant effect on plans for opioid disposal and how seriously respondents viewed issues of opioid misuse. When asked their preferred location for prescription opioid disposal, the most common preference was a local pharmacy.

Discussion

This survey identified that most patients do not store their prescription opioids in a locked location, claim to dispose of their unused prescription opioids, and would prefer to dispose of them at a pharmacy if possible. This information points to the need for close prescriber-to-pharmacy collaboration to promote the safe disposal of prescription opioids and mitigate drug diversion.

## Introduction

The opioid epidemic has pushed prescribers to widely reassess prescribing practices to curb community opioid availability and opioid-related deaths. Critical to this discussion are patterns and behaviors related to opioid disposal, as drug diversion and accidental ingestion of leftover prescriptions have been identified as major contributors to opioid misuse. The Substance Abuse and Mental Health Services Administration reported in 2017 that 50.5% of people who misused prescription pain relievers received their most recent pain relievers from a friend or relative [1]. The problems posed by drug diversion have prompted a variety of public health strategies to improve drug disposal. Community interventions typically involve public education campaigns highlighting the dangers of prescription pain relievers and/or community drug take-back programs promoting the safe disposal of leftover medications [2]. In 2014, the U.S. Drug Enforcement Administration (DEA) implemented the Secure and Responsible Drug Disposal Act, expanding options for controlled substance collection sites to include pharmacies, hospitals, clinics with onsite pharmacies, and narcotics treatment programs [3-4]. Still, inadequate disposal of unused medications remains a significant part of the opioid crisis, and solutions to improve disposal are urgently required.

Prior studies suggest that lack of patient education may be a significant contributor to low rates of appropriate opioid disposal. A study of pharmacists found that a majority provided medication disposal education once a month or less, and only 10.1% of the pharmacists in the study could accurately identify all appropriate recommendations for controlled substance disposal [5]. Disposal education is provided at a similarly low rate by surgeons in the perioperative setting [6-9]. In studies where postoperative medication disposal statistics are reported, disposal rates in the postoperative period are low across several surgeries. A systematic review of studies across seven types of surgery found postoperative disposal rates between 4% (C-section, thoracic surgery) and 30% (dental surgery) while only 4-9% of patients disposed of medications in a manner consistent with Food and Drug Administration (FDA) recommendations [10].

Given the low rate of education, prior interventional studies have attempted to assess whether increased education in the perioperative setting may improve patient drug disposal behavior. Educational strategies employed in these studies included disposal instruction sheets, information about pharmacy-based disposal programs, and disposal education by nursing staff [11-12]. One individual randomized trial suggested there may be a benefit to additional perioperative education; however, education resulted in only modest increases in postoperative opioid disposal [12]. Moreover, when randomized control trials were considered together, a meta-analysis demonstrated a reduction in overall opioid consumption but with no significant difference in drug disposal when perioperative education was employed [13]. The results point to the potential for an unintended increase in unused opioids available for diversion when opioid consumption is mitigated without a concomitant reduction in disposal.

While there may be a role for opioid education in the perioperative setting, studies have thus far been unable to produce more than modest improvements in disposal rates. These findings suggest that individual patient attitudes may be driving disposal behaviors to a greater degree than lack of education. However, correlations between individual patient factors and postoperative opioid disposal have not been well-described in prior literature. The present study sought to determine disposal preferences in patients receiving postoperative opioid prescriptions following orthopedic surgery and to identify predictors of individual opioid disposal behaviors.

## Materials and methods

A cross-sectional survey of patients following orthopedic surgeries in a variety of subspecialties was used to determine prescription opioid storage and disposal behaviors in the postoperative setting. Patients who received surgery at a single, large academic orthopedic surgery practice from November 2021 to March 2022 were recruited to participate in the study during their first postoperative visit. Patients who agreed to complete the survey were presented with a digital quick response (QR) code or website address and instructions on how to access a web-based survey using their personal electronic devices. Enrollment in the study was limited to patients who were able to access the survey via an electronic device at their first postoperative visit.

The online survey consisted of 12 questions regarding patient-specific characteristics including the type of surgery, age, gender, whether a postoperative opioid prescription was received, attitudes toward prescription drug misuse, and methods for storage and disposal of prescription opioids (see Appendix). Upon survey completion, respondents were taken to a final screen prompting them to submit the survey. Alternatively, the survey was ended early if a respondent indicated they did not receive an opioid prescription for their surgery. Surveys submitted with all 12 questions completed were included in the statistical analysis. Surveys from respondents who indicated they did not receive a prescription opioid were excluded from the analysis. The primary outcome was a self-reported preference for the location of opioid disposal. Secondary outcomes included self-reported opioid storage and disposal behaviors.

These data were first processed to reconcile written responses when “Other” was selected in relevant survey questions. Irrelevant data from written responses were additionally excluded from analysis, and the survey data was tabulated to characterize overall response trends. The most common preference for the location of opioid disposal was determined via the percentage of total selections for each disposal location.

These data were subsequently analyzed using chi-squared tests to determine associations between participant characteristics and survey responses. Subgroup analyses by participant gender and age were conducted for four of the survey questions: 1) whether participants kept unused pain medications, 2) whether participants were concerned about others using their pain medication, 3) how seriously participants viewed prescription drug misuse, and 4) participants' plans for the disposal of unused pain medication. Associations with a p-value of <0.05 were considered statistically significant.

## Results

**Figure 1:**
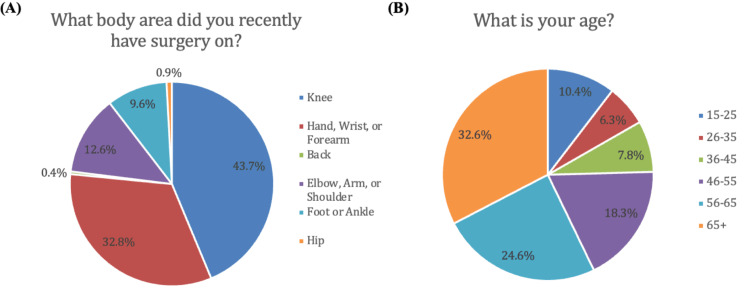
Demographic characteristics of 469 initial survey respondents

**Figure 2:**
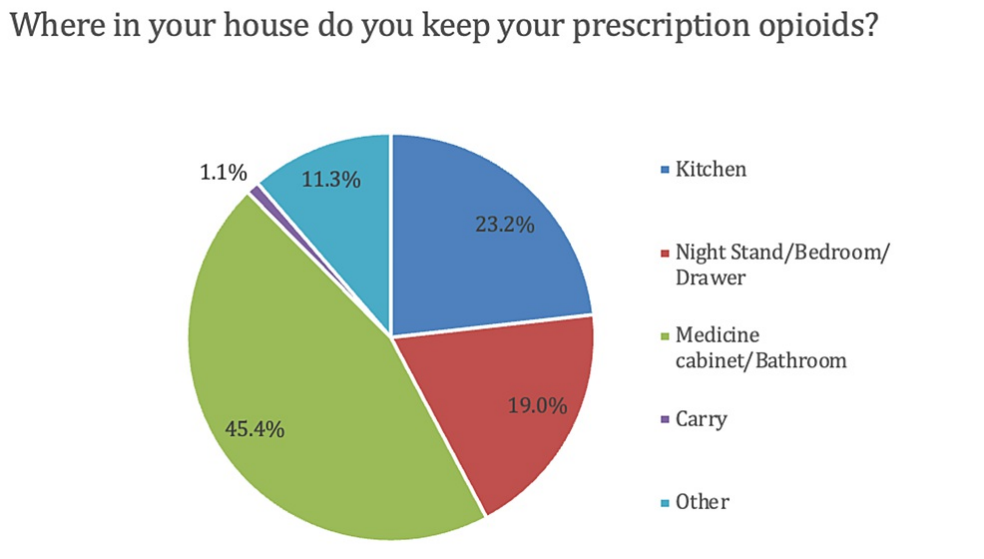
Locations where participants stored postoperative prescription opioids

**Figure 3:**
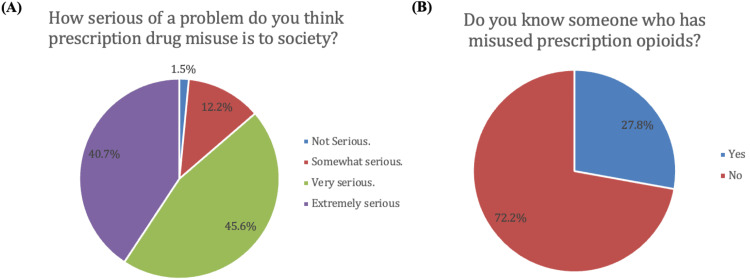
Respondent attitudes towards prescription drug misuse

A total of 469 patients responded to the survey. The demographic characteristics of respondents are represented in Figure 1. Surgeries represented included procedures on the knee, hand/wrist/forearm, spine, elbow/arm/shoulder, foot/ankle, and hip (Figure 1A). Among initial respondents, 229 (48.8%) were female, 240 (51.2%) were male, and the majority of patients (75.5%) were ≥46 years old (Figure 1B). Of the initial respondents, 74 (15.8%) ­patients indicated they did not receive a postoperative opioid prescription and were excluded from statistical analysis, leaving 395 patients available for analysis. Regarding postoperative storage of opioids, 342 respondents (86.6%) reported storing their opioids in an unlocked location. The most common locations for opioid storage included the bathroom (45.4%), kitchen (23.2%), bedroom/nightstand (19.0%), or another location (11.3%) (Figure 2).When asked how serious of a problem they thought prescription drug misuse was to society, 161 participants (40.7%) felt it was extremely serious, 180 (45.6%) felt it was very serious, 48 (12.2%) felt it was somewhat serious, and six (1.5%) felt it was not serious (Figure 3A). Additionally, 110 respondents (27.8%) knew somebody who had misused prescription drugs while 285 (72.2%) did not (Figure 3B).

**Figure 4:**
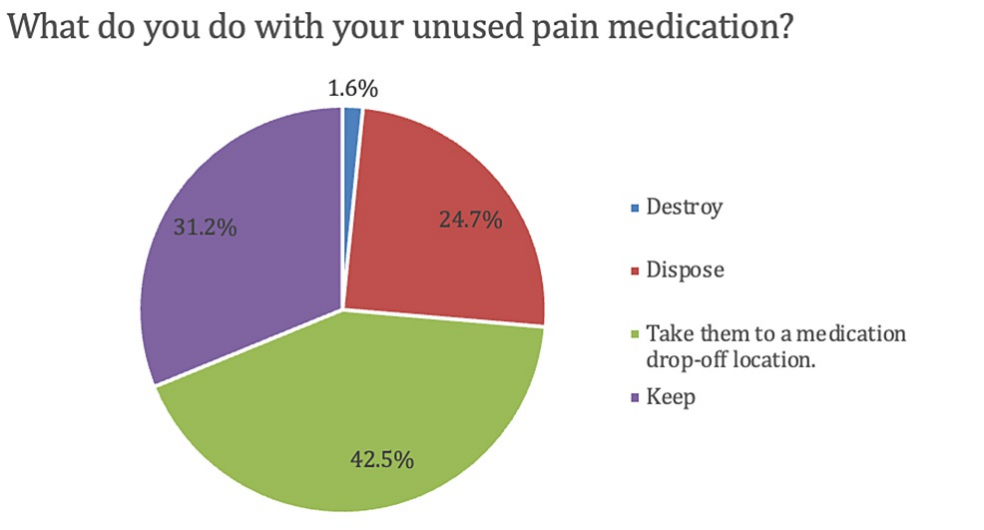
Actions taken with unused medications

Of those who were prescribed opioids, 372 participants (94.2%) reported having leftover unused prescription opioids after their surgery. A total of 256 participants with leftover opioids (68.8%) reported disposing of their unused opioids (Figure 4). The most commonly indicated preference for the site of opioid disposal was a local pharmacy (77.4% of respondents), followed by the police department, physician's office, fire station, special community collection event, and fire station (Table 1).

**Table 1:** Preferred sites for disposal of unused opioids

Preferred Site	Percent of patients (N = 372)
Local pharmacy	77.4%
Physician’s office	29.6%
Police department	32.3%
Fire station	14.8%
Municipal hazardous waste facility	8.3%
Special community collection event	22.3%
Other	4.6%

Subgroup analysis by gender revealed a statistically significant difference between males and females regarding how seriously participants viewed prescription drug misuse (P=0.035) and plans for disposal of medication (P=0.009). No statistical significance was observed in the effects of gender on responses to other survey questions (Table 2). Subgroup analysis by age similarly did not reveal statistically significant effects across all studied questions (Table 3).

**Table 2:** Associations between respondent gender and responses to survey questions

Question/Response Choices	Female (N = 186)	Male (N = 209)	P-Value
Kept their prescription opioids?			0.107
No	167 (89.8%)	175 (83.7%)	
Yes	19 (10.2%)	34 (16.3%)	
Concerned about children, family members, or friends using the respondent’s pain medication?			0.706
No	177 (95.2%)	196 (93.8%)	
Yes	9 (4.84%)	13 (6.22%)	
How serious of a problem did they consider misuse of prescription drugs?			0.035
Not Serious	1 (0.54%)	5 (2.39%)	
Somewhat Serious	23 (12.4%)	25 (12.0%)	
Very Serious	74 (39.8%)	106 (50.7%)	
Extremely Serious	88 (47.3%)	73 (34.9%)	
Disposal plan for their prescription opioids?	N = 178	N = 193	0.009
Disposed	35 (19.7%)	56 (29.0%)	
Destroyed	5 (2.81%)	1 (0.52%)	
Dropped Off	88 (49.4%)	70 (36.3%)	
Kept	50 (28.1%)	66 (34.2%)	

**Table 3:** Associations between respondent age and responses to survey questions

Question/Response Choices	Ages 18-25 (N=47)	Ages 26-35 (N=27)	Ages 36-45 (N=29)	Ages 46-55 (N=76)	Ages 56-65 (N=93)	Ages 65+ (N=114)	P-value
Kept their prescription opioids?							0.942
No	40 (85.1%)	23 (85.2%)	26 (89.7%)	64 (84.2%)	81 (87.1%)	101 (88.6%)	
Yes	7 (14.9%)	4 (14.8%)	3 (10.3%)	12 (15.8%)	12 (12.9%)	13 (11.4%)	
Concerned about children, family members, or friends using the respondent’s pain medication?							0.694
No	44 (93.6%)	25 (92.6%)	29 (100%)	73 (96.1%)	86 (92.5%)	108 (94.7%)	
Yes	3 (6.38%)	2 (7.41%)	0 (0.00%)	3 (3.95%)	7 (7.53%)	6 (5.26%)	
How serious of a problem did they consider misuse of prescription drugs?							0.103
Not Serious	0 (0.00%)	0 (0.00%)	1 (3.45%)	2 (2.63%)	1 (1.08%)	2 (1.75%)	
Somewhat Serious	8 (17.0%)	3 (11.1%)	5 (17.2%)	14 (18.4%)	9 (9.68%)	8 (7.02%)	
Very Serious	15 (31.9%)	11 (40.7%)	13 (44.8%)	30 (39.5%)	40 (43.0%)	68 (59.6%)	
Extremely Serious	24 (51.1%)	13 (48.1%)	10 (34.5%)	30 (39.5%)	43 (46.2%)	36 (31.6%)	
Disposal plan for their prescription opioids?	(N=42)	(N=25)	(N=28)	(N=69)	(N=88)	(N=108)	0.592
Disposed	15 (35.7%)	8 (32.0%)	8 (28.6%)	15 (21.7%)	20 (22.7%)	24 (22.2%)	
Destroyed	0 (0.00%)	0 (0.00%)	1 (3.57%)	2 (2.90%)	2 (2.27%)	0 (0.00%)	
Dropped Off	17 (40.5%)	13 (52.0%)	10 (35.7%)	30 (43.5%)	38 (43.2%)	44 (40.7%)	
Kept	10 (23.8%)	4 (16.0%)	9 (32.1%)	22 (31.9%)	28 (31.8%)	40 (37.0%)	

## Discussion

The present study found that the majority (68.8%) of patients with unused opioids reported disposing of them by several different methods, including destruction, personal disposal, and returning to a drop-off location for unused medications. Gender had a significant effect on plans for opioid disposal and on how seriously participants viewed issues of prescription drug misuse. A greater proportion of females than males disposed of opioids (71.9% of females vs. 65.8% of males) and considered opioid misuse a very or extremely serious issue (87.1% females vs. 85.6% males). These findings point to a greater sensitivity to opioid-related issues in females, which aligns with prior studies demonstrating women are less likely than men to report opioid misuse [14]. This may indicate the need for gender-specific interventions though future studies should qualify gender associations in regards to opioid use to best inform these interventions.

When asked about preferences for a potential opioid disposal location, more participants preferred to use a local pharmacy. A prior study on prescription drug disposal suggested patients considered pharmacies the most convenient location for drug disposal; however, the study was limited by convenience sampling of pharmacy patrons attending a pharmacy-based drug take-back event [15]. Despite these limitations, the present study similarly found that patients consider pharmacies most convenient for drug disposal. This suggests an important role for pharmacies in increasing drug disposal after surgery. However, current literature suggests opioid disposal education may not be delivered consistently by pharmacists [5]. Given patient preferences for pharmacy-based disposal, efforts to improve pharmacy-based interventions, including point-of-service education regarding proper handling and disposal of unused opioids, may be essential in promoting drug disposal. Additionally, as opioid prescribers, surgeons have an important role in improving drug disposal among their patients. Although efforts have been made to curb opioid overprescribing following surgery, surgeons may not be pairing this with appropriate discussion of opioid disposal [6-9]. Continued patient education across the course of care, including pre and postoperative education by surgeons and pharmacists, will be essential to ensuring appropriate opioid disposal by patients and an overall reduction in community opioid availability.

A large proportion of our study participants indicated they disposed of their prescription opioids. While the nature of the study question risks overreporting and social-desirability bias, the results may reflect a high baseline community awareness of opioid-related issues. Future studies must consider regional variation in community knowledge since certain interventions (e.g., education) may not be impactful for all populations. Results from this single-center study are unlikely to account for regional variations in education and socioeconomic status and may not be generalizable across populations in the United States. Interpretation of the survey was limited due to its administration to patients receiving a range of surgeries. Depending on the procedure performed, postoperative courses and patient satisfaction with pain control would differ widely at the first postoperative visit, which may have adversely skewed survey responses. Additionally, data on the type and number of opioids prescribed/consumed were not collected, limiting the study’s ability to draw conclusions regarding opioid use and its impact on disposal in the study population. Importantly, while participants had a high rate of unused opioids, the study did not determine whether overprescribing was a factor driving this trend. Future studies on postoperative drug disposal should evaluate pain scores and quantify opioids prescribed/consumed to best distinguish factors influencing drug disposal behavior.

Future studies should seek to qualify the association between gender and opioid disposal. Additionally, studies on postoperative pain management may consider protocols that include pharmacy-based drug disposal to evaluate the utility of multipronged approaches to reduce opioid consumption.

This study provides valuable data to optimize efforts to promote drug disposal. During discrete periods of opioid use such as the postoperative setting, there are routine opportunities for active drug disposal intervention. Patients returning to pharmacies for prescription renewal or acquisition of step-down medication may be targets for these interventions. Importantly, multimodal pain management strategies are proving effective in reducing postoperative opioid consumption in the orthopedic setting [16]. When combined with a multimodal strategy, point-of-service disposal interventions may further mitigate the risk of diversion of unused opioids. Focusing efforts on the postoperative patient population will help ensure healthcare resources are optimized for impact without overburdening the system. Certainly, better provider-to-pharmacy collaboration will be critical to promoting disposal and protecting the community from drug diversion.

## Conclusions

Diversion of unused prescription opioids after surgeries is a common source of opioid sensitization and misuse. Individual patient attitudes may be driving opioid disposal behaviors to a greater degree than lack of education on safe opioid use. Our study found that most patients do not store their prescription opioids in a locked location, dispose of their unused prescription opioids, and prefer to dispose of them at a pharmacy if possible. This suggests a need for close prescriber-to-pharmacy collaboration to promote the safe disposal of prescription opioids and mitigate diversion. Further studies considering regional variations in community education and individual pain scores are needed.

## Appendices

**Table 4:** Opioid disposal online survey questionnaire

Q1: What body area did you recently have surgery on?
Neck
Back
Hand, wrist, or forearm
Elbow, arm, or shoulder
Hip
Knee
Foot or ankle
Q2: What is your age?
18-25
26-35
36-45
46-55
56-65
65+
Q3: Describe your gender.
Male
Female
Other: ________
Q4: Were you prescribed opioids (ie, oxycodone, Percocet, Vicodin, etc) after surgery?
Yes
No
Q5: Where in your house do you keep your prescription opioids?
Describe: _______
Q6: Is this location locked?
Yes
No
Q7: What do you do with your unused pain medication?
I hold on to them
Flush them down the toilet
Throw them in the trash
Take them to a medication drop-off location
Other: _______
Q8: Are you concerned about any children, family members, or friends using your pain medication?
Yes
No
Q9: Do you know someone who has misused prescription opioids?
Yes
No
Q10: How serious of a problem do you think prescription drug misuse is to society?
Not serious
Somewhat serious
Very serious
Extremely serious
Q11: Have you ever attended a drug take-back event?
Yes
No
Q12: Where would be best for you to dispose of any opioids? Choose as many as you like.
At a local pharmacy
At my physician’s office
At a special collection event in my community
At a police department
At a fire station
At a municipal hazardous waste facility
Other: _______
